# Suspected Type II Kounis Syndrome with Coronary Dissection Extending from the Distal Left Main into the Proximal Left Anterior Descending Artery After Wasp Sting Anaphylaxis: A Case Report

**DOI:** 10.3390/jcdd13100509

**Published:** 2026-10-10

**Authors:** Ivans Mirzojans, Irina Holste, Adīne Kaņepa, Alberts Bērziņš

**Affiliations:** 1 Faculty of Medicine, Rīga Stradiņš University, 16 Dzirciema iela, 1007 Rīga, LV, Latvia; 2 Department of Invasive Cardiology, Riga East Clinical University Hospital, Hipokrāta iela 2, 1079 Rīga, LV, Latvia; 3 Clinical Centre of Immunology and Allergology, Pauls Stradiņš Clinical University Hospital, Pilsoņu iela 13, 1002 Rīga, LV, Latvia

**Keywords:** Kounis syndrome, anaphylaxis, coronary dissection, intravascular ultrasound, case report

## Abstract

Kounis syndrome is an acute coronary syndrome occurring in temporal association with an allergic or anaphylactic reaction. Recognition may be particularly challenging in patients with established coronary artery disease, in whom hypersensitivity-mediated mechanisms may coexist with conventional causes of acute myocardial infarction. We report a 62-year-old man with known coronary artery disease and previous percutaneous coronary interventions who developed loss of consciousness, generalized urticaria, facial swelling, dyspnea, and hemodynamic instability shortly after two wasp stings. He received intramuscular adrenaline and supportive treatment for anaphylaxis. Severe chest pain subsequently developed, with ST-segment elevation and a dynamic increase in cardiac troponin. Emergency coronary angiography demonstrated a critical lesion extending from the distal left main coronary artery into the proximal left anterior descending artery. Intravascular ultrasound performed before stent implantation demonstrated an intimal flap and dissection plane. Intravascular ultrasound-guided percutaneous coronary intervention with drug-eluting stent implantation achieved a satisfactory acute result. Subsequent allergological evaluation demonstrated Hymenoptera venom sensitization; baseline serum tryptase measured approximately five weeks after the event was within the reference range. The close temporal relationship between anaphylaxis and myocardial infarction supports suspected type II Kounis syndrome, while coronary dissection was identified as the culprit lesion morphology; its causal relationship to the hypersensitivity reaction remains uncertain.

## 1. Introduction

Kounis syndrome describes the occurrence of acute coronary syndrome in temporal association with an allergic, hypersensitivity, or anaphylactic reaction. Activation of mast cells and other inflammatory pathways may result in the release of histamine, tryptase, leukotrienes, platelet-activating factor, and other mediators capable of inducing coronary vasomotor abnormalities, platelet activation, thrombosis, and destabilization of pre-existing atherosclerotic lesions [1,2].

Three principal variants are commonly recognized. Type I occurs in patients without established coronary artery disease and is predominantly associated with coronary vasospasm, with or without myocardial injury. Type II occurs in patients with pre-existing atherosclerotic disease in whom an allergic event may precipitate vasospasm, plaque destabilization, or acute myocardial infarction. Type III is associated with coronary stent thrombosis in the setting of hypersensitivity [1].

Hymenoptera venom is a recognized trigger of Kounis syndrome. More broadly, reported triggers include medications, particularly antibiotics and nonsteroidal anti-inflammatory drugs, as well as iodinated contrast media, anesthetic and perioperative agents, antineoplastic drugs, foods, and environmental exposures [1,2]. Published cases following bee or wasp stings have demonstrated a heterogeneous spectrum of coronary pathology, including vasospasm, intracoronary thrombosis, plaque-related acute myocardial infarction, and complete coronary occlusion [3,4]. Coronary artery dissection has been described considerably less frequently. A previous report documented proximal left anterior descending artery dissection after multiple honeybee stings in a young patient without established coronary artery disease [5].

The present case is noteworthy because clinically evident anaphylaxis after wasp stings was followed by ST-segment elevation myocardial infarction in a patient with extensive pre-existing coronary artery disease, while intravascular ultrasound performed before definitive coronary intervention demonstrated a dissection extending from the distal left main coronary artery into the proximal left anterior descending artery. The case illustrates both the diagnostic complexity of suspected type II Kounis syndrome and the value of intracoronary imaging in defining the mechanism of an angiographically complex culprit lesion.

## 2. Case Report

A 62-year-old man with established multivessel coronary artery disease, arterial hypertension, hypercholesterolemia, chronic heart failure with preserved left ventricular systolic function, and type 2 diabetes mellitus was stung twice by a wasp in the left shoulder region at approximately 19:25 on 23 June 2026.

His coronary history included staged multivessel percutaneous coronary intervention in 2024: PCI of the LCx with drug-eluting stent implantation on 17 June 2024; PCI of the LAD with drug-eluting stent implantation and adjunctive plain balloon angioplasty on 29 July 2024; and PCI of the RCA with drug-eluting stent implantation on 2 October 2024. At the index angiography in June 2026, the previously implanted proximal LAD, LCx/OM3, and mid-RCA stents were patent without angiographic restenosis. No previous left main PCI or documented left main lesion was identified in the available records. Exact dimensions of the 2024 stents and lesion-level details of residual coronary disease after those procedures were not available in the source documentation reviewed for this report.

Baseline transthoracic echocardiography on 17 June 2024 showed preserved left ventricular ejection fraction of 60%, left ventricular hypertrophy, left atrial dilatation, mild mitral and tricuspid regurgitation, and no evidence of pulmonary hypertension (estimated right ventricular systolic pressure 25 mmHg). Following the October 2024 RCA PCI, the documented chronic regimen included clopidogrel/acetylsalicylic acid 75/100 mg once daily for 6 months followed by acetylsalicylic acid 100 mg once daily, perindopril/amlodipine 7/5 mg once daily, metoprolol succinate 50 mg once daily, atorvastatin 80 mg once daily, and continuation of antidiabetic therapy; pantoprazole 20 mg once daily was prescribed for 6 months. At the June 2026 index event, the prehospital history recorded that the patient was taking several medications but could not name them; therefore, the exact chronic regimen and medication adherence immediately before the event could not be independently verified. The patient had experienced wasp and other Hymenoptera stings previously, resulting only in mild local reactions. No other allergies were reported in the history.

Shortly after the stings, he developed a rapidly progressive systemic reaction characterized by generalized urticaria, facial swelling, dyspnea, loss of consciousness, and hemodynamic instability. The presentation was clinically consistent with anaphylaxis. Emergency treatment included intramuscular adrenaline 0.5 mg, repeated once because of an insufficient clinical response to the first dose, together with dexamethasone and intravenous fluid resuscitation.

**Figure 1:**
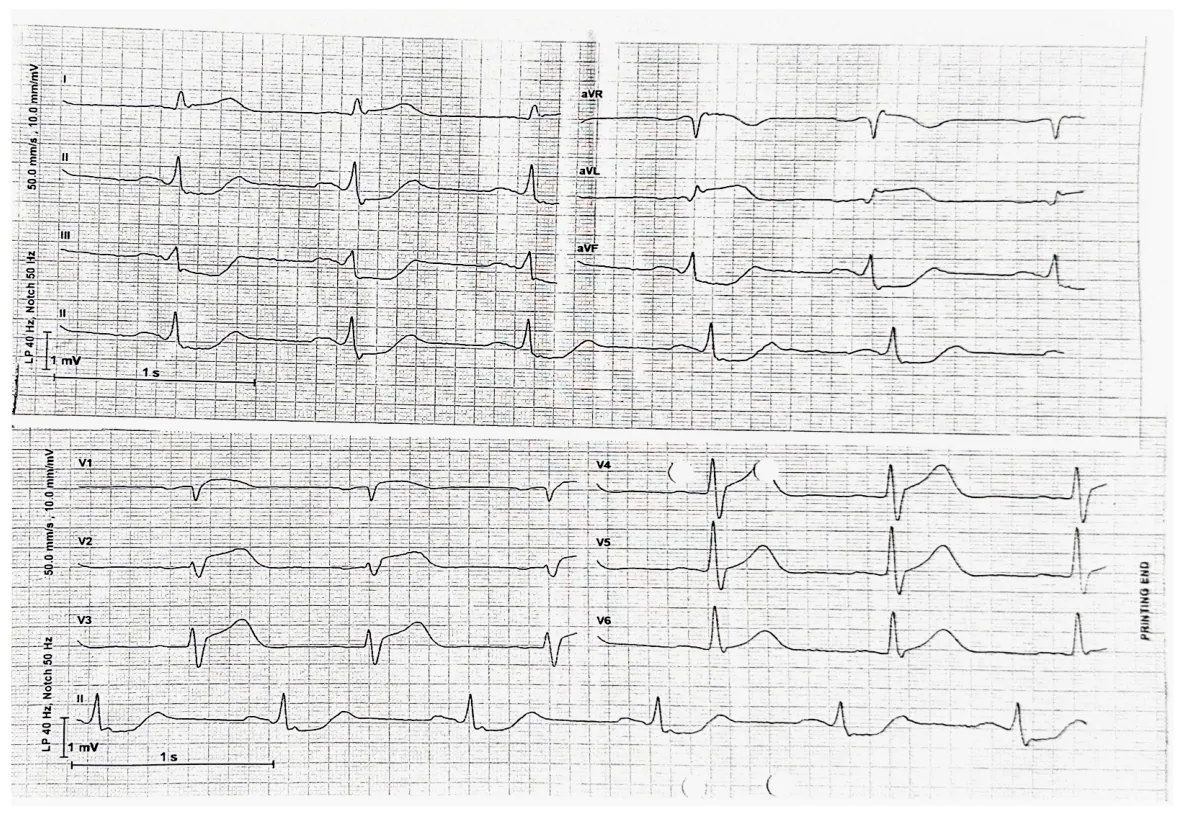
Prehospital electrocardiogram demonstrating extensive acute ischemic changes with ST-segment elevation in the anterior and lateral leads and reciprocal inferior ST-segment depression.

Following initial treatment of the allergic reaction, the patient developed severe pressing chest pain. Electrocardiography demonstrated acute ischemic changes with ST-segment elevation. Serial high-sensitivity cardiac troponin T measurements showed a dynamic elevation consistent with acute myocardial injury, and urgent invasive evaluation was initiated (Figure 1). The reconstructed clinical sequence, including vital signs and treatment, is summarized in Table 1.

Laboratory investigations during the acute admission did not demonstrate eosinophilia. Monocytosis was present. Given its nonspecific nature in the setting of acute systemic stress and myocardial infarction, this finding was not considered evidence of an allergic coronary mechanism.

Medical management for acute coronary syndrome included aspirin, ticagrelor, intravenous unfractionated heparin, and morphine. Hemodynamic support was provided according to the patient’s clinical condition.

**Figure 2:**
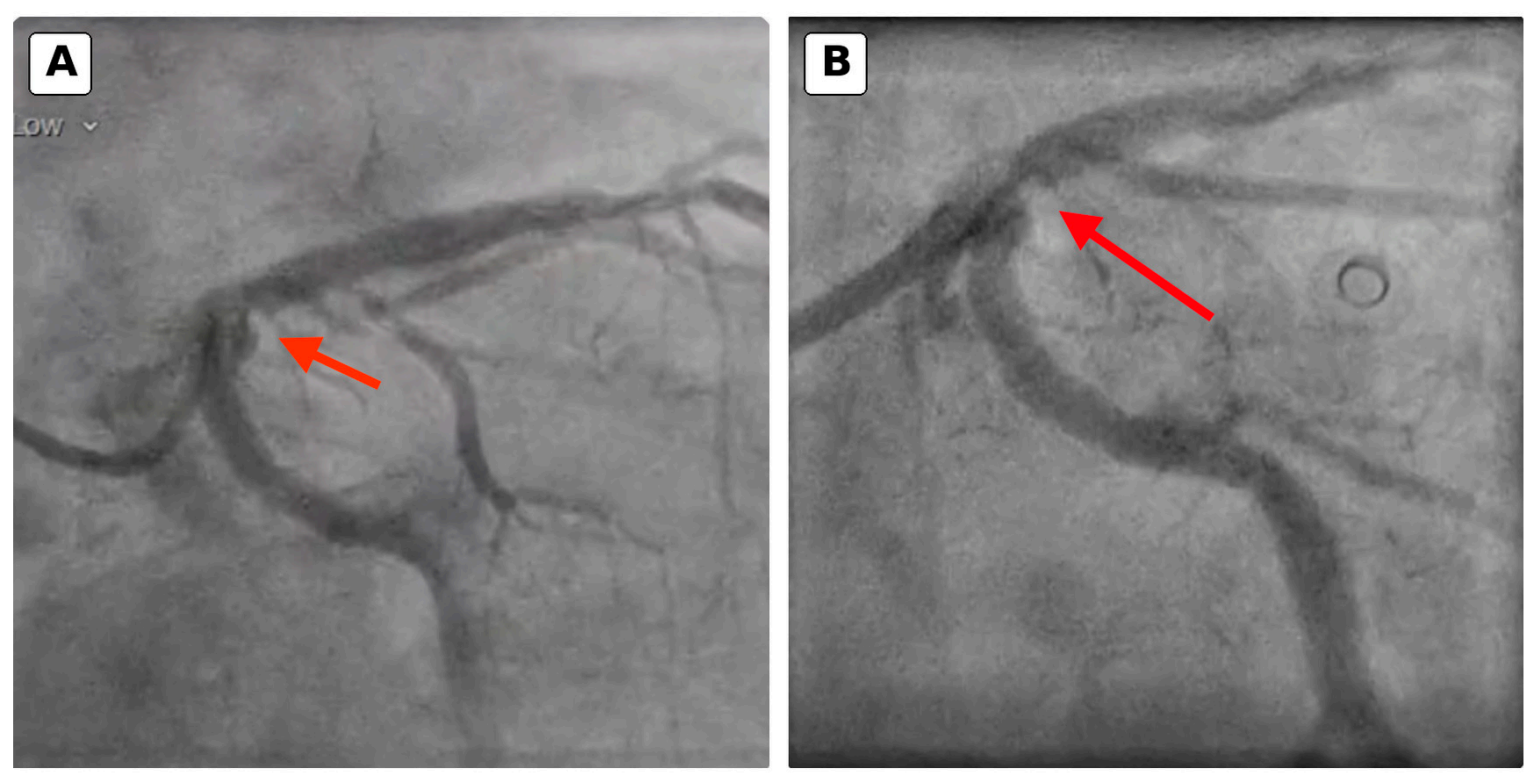
Initial coronary angiography before percutaneous coronary intervention. Panels (**A**,**B**) show two projections of the left coronary system obtained on the initial angiographic acquisition, before passage of any coronary guidewire and before IVUS, balloon dilatation, or stent implantation. The red arrows in panels (**A**,**B**) indicate the distal left main to proximal LAD culprit segment, which was already abnormal at this stage; subsequent IVUS demonstrated an intimal flap and dissection plane within this segment. The complete angiographic sequence, including right coronary angiography and the final post-PCI result, is provided as Supplementary Video S1.

Emergency coronary angiography demonstrated significant pre-existing coronary atherosclerosis and a critical culprit lesion involving the distal left main coronary artery and proximal left anterior descending (LAD) artery (Figure 2). The initial left coronary angiographic acquisitions shown in Figure 2 were obtained before passage of any coronary guidewire and before IVUS, balloon dilatation, or stent implantation. The culprit abnormality involving the distal left main to proximal LAD was therefore already present on the first angiographic acquisition. The available procedure report documents use of a 7 Fr EBU 3.75 guide catheter and does not record pressure damping, ventricularization, deep guide catheter seating, or another catheter-related complication. The complete initial and final left coronary angiographic sequences are provided as Supplementary Video S1.

**Figure 3:**
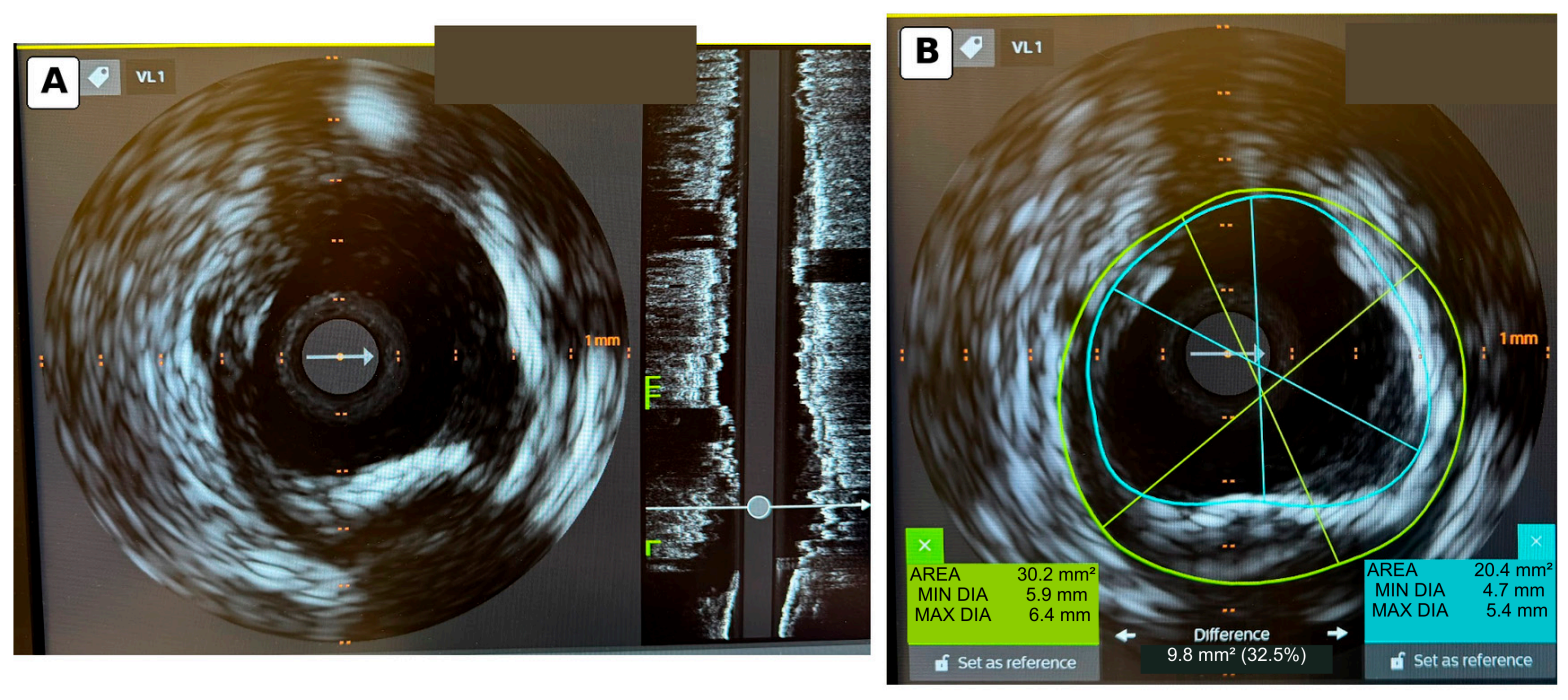
Pre-intervention intravascular ultrasound of the distal left main to proximal LAD culprit segment. Panel (**A**) shows a representative cross-sectional IVUS image of the culprit segment, in which an intimal flap and dissection plane were identified during the examination. Panel (**B**) shows the green and cyan planimetric contours with their corresponding diameter lines and measurement readouts. The central pale blue arrows and the markers in the longitudinal display are console interface indicators, not annotations of the dissection; the orange ticks indicate the distance scale. These are representative static frames; the exact proximal and distal boundaries of the dissection and its precise relationship to the previously implanted proximal LAD stent cannot be determined reliably from the available still images alone. The complete available IVUS recording is provided as Supplementary Video S2.

Intravascular ultrasound (IVUS) was performed to characterize the lesion before definitive stent implantation. The examination demonstrated an intimal flap and dissection plane involving the distal left main to proximal left anterior descending artery segment. Because these findings were documented before stent deployment, the dissection was present before definitive percutaneous treatment (Figure 3). After the initial angiographic acquisition, a soft coronary guidewire was advanced across the LM to LAD lesion, a second guidewire was positioned in the LCx, and IVUS was then performed. Predilatation and stent implantation occurred only after IVUS characterization of the lesion. The representative still frames illustrate the culprit morphology, but the exact proximal and distal dissection boundaries cannot be reconstructed from static images alone. The available IVUS recording from the index procedure is provided as Supplementary Video S2.

**Figure 4:**
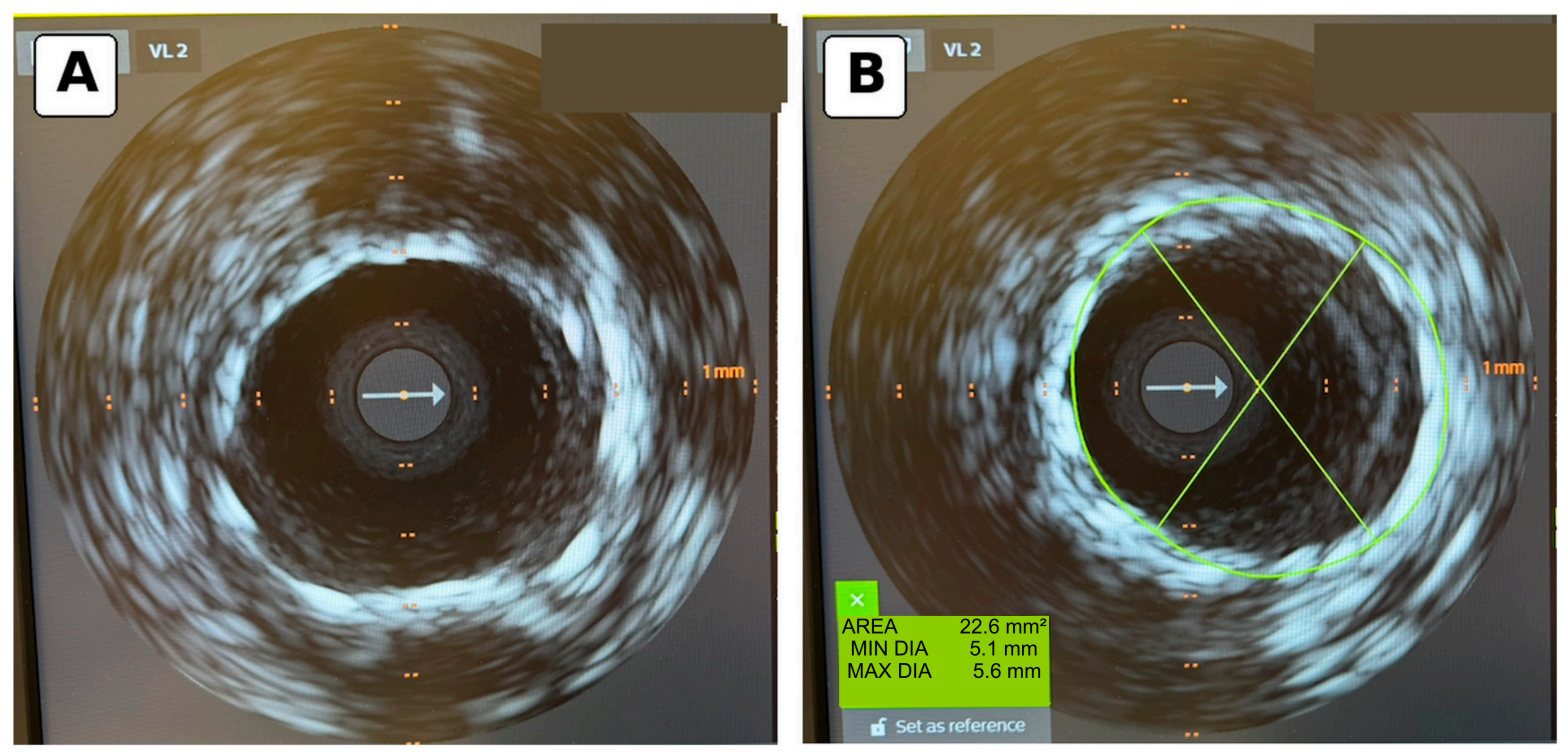
Post-intervention intravascular ultrasound after left main to LAD stent implantation. Panels (**A**,**B**) demonstrate the treated segment, including the post-PCI stent lumen and stent contour. The central pale blue arrows are console interface indicators, not annotations of residual dissection. The green contour and crossing lines in panel (**B**) show the selected planimetric measurement and diameters; orange ticks indicate the distance scale. In Panel (**B**), the IVUS console displays an area of 22.6 mm^2^, a minimum diameter of 5.1 mm, and a maximum diameter of 5.6 mm. No obvious residual dissection is visible in the selected frame. Systematic assessment of stent apposition, percentage stent expansion, and proximal and distal stent edges cannot be reconstructed reliably from the available archived static images.

**Figure 5:**
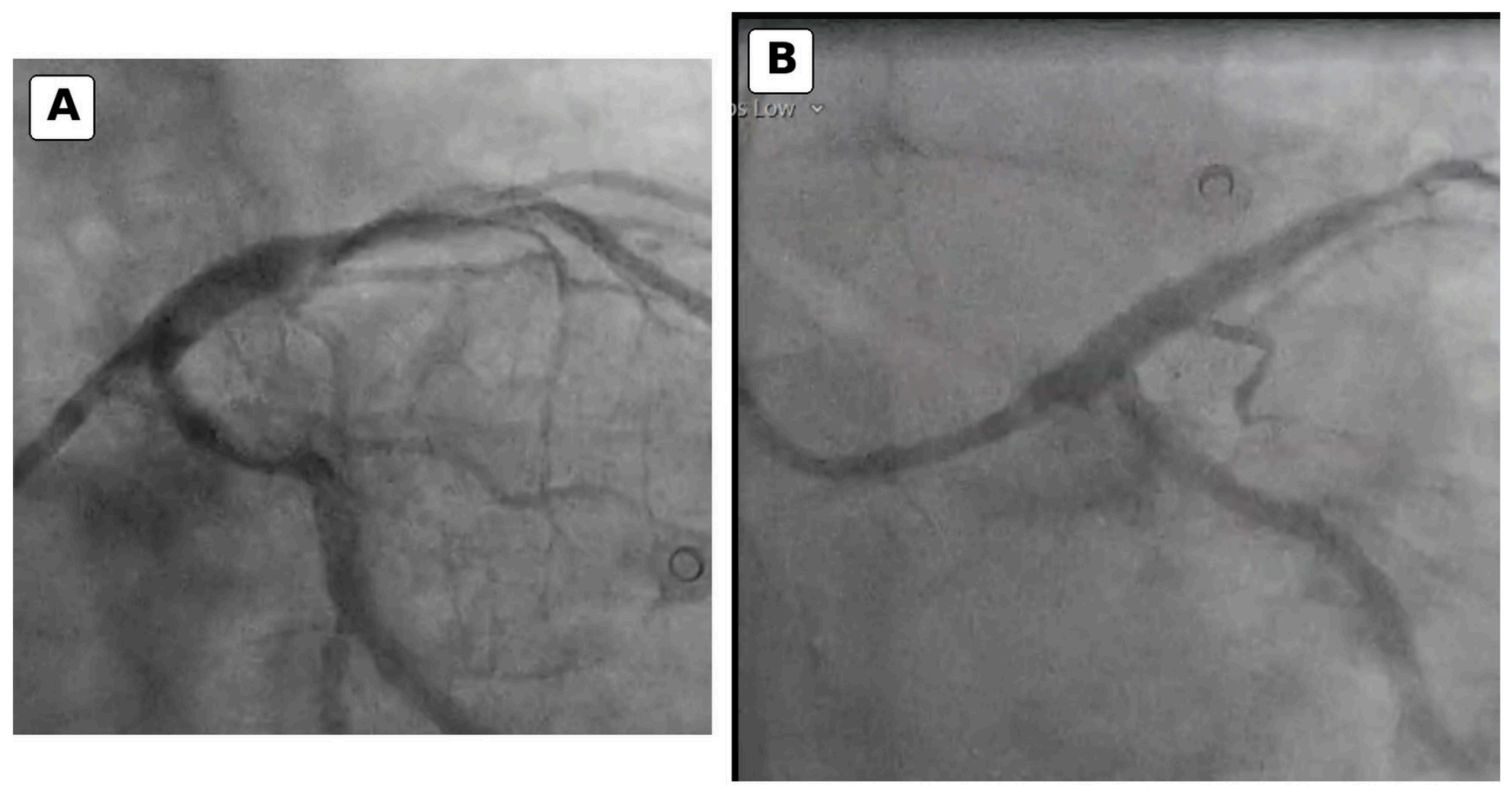
Final coronary angiography after IVUS-guided percutaneous coronary intervention from the left main toward the LAD. Panels (**A**,**B**) demonstrate a patent treated distal left main to proximal LAD segment with preserved LCx opacification and flow after drug-eluting stent implantation. The final angiographic sequence is included in Supplementary Video S1.

Intravascular ultrasound-guided percutaneous coronary intervention was subsequently performed, with implantation of a Xience Skypoint 5.0 × 15 mm drug-eluting stent from the left main toward the left anterior descending artery. Final angiographic assessment demonstrated a satisfactory acute result, while the available post-intervention IVUS frame documented the treated segment. In the available post-intervention IVUS frame, the measured area was 22.6 mm^2^, with a minimum diameter of 5.1 mm and a maximum diameter of 5.6 mm (Figure 4). This value represents the selected archived frame and is not presented as a pullback-derived minimum stent area. A formal percentage stent expansion and quantitative assessment of both stent edges cannot be reconstructed from the available static images. No obvious residual dissection is visible in the selected frame. Systematic assessment of stent apposition cannot be performed reliably from the available archived static images. Final angiography demonstrated preserved LCx flow after left main to LAD stenting (Figure 5). The exact degree of overlap between the newly implanted left main to LAD stent and the previously implanted proximal LAD stent cannot be determined reliably from the available still images.

Following revascularization, the patient’s chest pain resolved. High-sensitivity cardiac troponin T increased from 23.2 ng/L on admission to 536 ng/L the following morning, followed by a decrease to 484 ng/L on 25 June 2026 and 247 ng/L at discharge. A previous value of 10.1 ng/L from 27 January 2026 was available for comparison.

Transthoracic echocardiography performed on 29 June 2026 demonstrated a left ventricular ejection fraction of 54%. Regional wall motion assessment showed hypokinesia of the mid-anteroseptal and peri-apical segments. The E/e′ ratio was 6.1, tricuspid annular plane systolic excursion was 19 mm, and estimated right ventricular systolic pressure was 25–30 mmHg. Mild mitral and tricuspid regurgitation were present. No pericardial effusion was observed.

After revascularization, no recurrent chest pain was documented. The available records document inpatient care from 23 June through at least 29 June 2026. During hospitalization, post-infarction monitoring and optimization of secondary prevention therapy were continued, and the patient was subsequently discharged in clinically stable condition with recommendations for continued cardiology and allergology follow-up. No later transthoracic echocardiography or cardiac magnetic resonance examination was performed during the available follow-up; consequently, subsequent LVEF, regional wall motion recovery, infarct extent, scar burden, and myocardial viability could not be assessed.

At hospital discharge, the antithrombotic regimen consisted of ticagrelor 90 mg twice daily through 23 June 2027 together with acetylsalicylic acid 100 mg once daily long-term, corresponding to 12 months of dual antiplatelet therapy after the index acute coronary syndrome and percutaneous coronary intervention. The remaining discharge treatment included rosuvastatin 40 mg once daily, perindopril/amlodipine 7/5 mg once daily, nebivolol 5 mg once daily, spironolactone 25 mg once daily, and pantoprazole 20 mg once daily while ticagrelor was continued. Diabetes treatment comprised vildagliptin/metformin 50/1000 mg twice daily and empagliflozin 10 mg once daily.

Allergological evaluation after discharge demonstrated Hymenoptera venom sensitization. On 28 July 2026, wasp venom-specific immunoglobulin E (IgE) was 0.41 kU/L and Ves v 5 specific IgE was 0.93 kU/L, whereas Ves v 1 was <0.350 kU/L. Whole bee venom-specific IgE was <0.350 kU/L; Api m 10 was 1.15 kU/L, while Api m 1 and Api m 2 were <0.350 kU/L. The laboratory reporting threshold for a positive specific IgE result was 0.35 kU/L. Ves v 5 is a major marker allergen for Vespula sensitization, and its positivity together with the clearly documented wasp sting supports clinically relevant vespid sensitization. However, antigen 5 allergens show substantial cross-reactivity within Vespidae, particularly between Vespula and Polistes species, so Ves v 5 alone does not establish the precise vespid species. Api m 10 is considered a marker of genuine honeybee sensitization and is not expected to cross-react with Ves v 5; isolated Api m 10 positivity in the setting of negative whole bee venom, Api m 1 and Api m 2 therefore suggests possible honeybee co-sensitization rather than proof of clinically relevant honeybee allergy. Component results were interpreted in the context of the sting history rather than in isolation [6]. Baseline serum tryptase was 5.3 µg/L (reference <8.4 µg/L). No acute tryptase sample had been obtained during the index reaction, so the later normal value cannot exclude acute mast cell activation.

At the subsequent allergy consultation, two 0.3 mg epinephrine auto-injector devices were prescribed, reflecting the locally available device strength. The patient and family members were instructed to administer 0.3 mg intramuscular epinephrine promptly into the anterolateral thigh for any recurrent systemic reaction, to repeat the dose after 5 to 10 min if symptoms failed to improve, and to activate emergency medical services. Bilastine 20 mg orally was prescribed for isolated mild cutaneous symptoms, and inhaled salbutamol 100 µg for persistent bronchospasm as adjunctive treatment; neither was intended to delay epinephrine in the event of a systemic reaction, respiratory compromise, or tongue or throat swelling. The allergist also recommended discussing substitution of the beta blocker with the treating cardiologist if clinically feasible. Given the severity of the index reaction and evidence of vespid sensitization, venom immunotherapy was recommended because current guidelines indicate venom immunotherapy after moderate to severe systemic sting reactions [7,8]. Venom immunotherapy was not withheld because of the patient’s coronary artery disease. After counseling regarding its preventive benefit, the patient declined initiation of venom immunotherapy. Current guidance does not regard stable cardiovascular disease, beta blocker therapy, or angiotensin-converting enzyme inhibitor therapy as absolute contraindications to venom immunotherapy [7,8]. The patient was advised to continue strict sting-avoidance measures, carry two epinephrine auto-injectors, and return for allergology follow-up within 6 to 12 months or earlier if another sting reaction occurs or if he wishes to reconsider venom immunotherapy. If he elects to proceed, treatment will be reconsidered jointly by allergology and cardiology with attention to cardiovascular stability, concomitant cardiovascular medication, and selection of the appropriate venom extract.

## 3. Discussion

The close temporal sequence of wasp sting anaphylaxis followed by objectively documented acute myocardial infarction supports suspected type II Kounis syndrome. No universally validated diagnostic criteria exist; in this case, the diagnosis rests on the convergence of a clinically convincing anaphylactic reaction, subsequent ischemic chest pain with transient extensive ST-segment elevation, a dynamic high-sensitivity cardiac troponin T rise, regional wall motion abnormalities, and a culprit coronary lesion requiring urgent PCI. The patient’s established multivessel atherosclerotic disease and previous PCI support classification as type II rather than type I Kounis syndrome [1,2,9,10].

Causality nevertheless remains inferential. Conventional plaque-related STEMI coinciding with anaphylaxis, adrenaline-associated ischemia, spontaneous coronary dissection, and an iatrogenic mechanism must all be considered. The culprit abnormality was visible on the initial left coronary angiographic acquisitions before any coronary guidewire passage, IVUS, balloon dilatation, or stent implantation, making injury from these subsequent devices unlikely. However, guide catheter engagement and the first contrast injection necessarily preceded the first angiographic frame. Because the available record cannot prove the vessel appearance before catheter engagement or contrast injection, a catheter- or injection-related contribution cannot be excluded completely. The dissection is therefore reported as the observed culprit morphology in the setting of suspected type II Kounis syndrome rather than as a proven hypersensitivity-mediated lesion.

A hypersensitivity-related contribution remains biologically plausible because mast cell mediators can alter coronary tone, platelet activity, coagulation, and atherosclerotic plaque stability [1,2,11]. Hymenoptera-associated Kounis syndrome has been reported with vasospasm, thrombosis, plaque-related infarction, and complete occlusion [3,4,12,13,14], whereas coronary dissection is uncommon. Del Rio Pertuz et al. described proximal LAD dissection after multiple honeybee stings [5]. The present case differs by the extensive pre-existing atherosclerotic burden and distal left main to proximal LAD involvement, but the sparse case-based evidence does not establish a direct mechanistic link between hypersensitivity and dissection. The clinical heterogeneity of Kounis syndrome is also illustrated by a recent report of type I disease characterized by recurrent vasospastic episodes rather than fixed structural coronary injury [15].

Multimodality imaging was useful for separating lesion morphology from its functional consequences. Angiography identified the complex distal left main to proximal LAD culprit lesion, IVUS directly demonstrated an intimal flap and dissection plane, and early transthoracic echocardiography documented an LVEF of 54% with regional hypokinesia. The early echocardiographic findings cannot be assumed to represent the final functional state because myocardial function may improve as stunned or viable myocardium recovers after acute injury and revascularization [16]. No later echocardiography or cardiac magnetic resonance imaging was performed, so infarct extent, scar burden, myocardial viability, and subsequent recovery of regional and global left ventricular function could not be assessed. A recent report by Gueli et al. illustrates the broader value of integrating angiography with non-invasive imaging to establish the mechanism of myocardial injury [17]. That report concerned MINOCA and is not directly comparable with the present case, which had obstructive coronary disease requiring PCI; it is cited here specifically for the mechanism-oriented multimodality imaging principle.

Adrenaline is another potential contributor that must be interpreted in context. The patient received two intramuscular doses of 0.5 mg because the response to the first dose was insufficient. Intramuscular adrenaline remains the first-line and potentially life saving therapy for anaphylaxis [9,10,18]. The systemic allergic reaction was already established before adrenaline administration, whereas chest pain was documented afterwards. This chronology does not identify adrenaline as the primary cause of the coronary event, although a contributory adrenergic or vasoconstrictive effect cannot be excluded.

The allergological findings support vespid sensitization but do not establish the coronary mechanism. Wasp venom-specific IgE and Ves v 5 were positive above the laboratory threshold of 0.35 kU/L, while the component profile also showed isolated Api m 10 positivity, compatible with possible honeybee co-sensitization rather than proven clinically relevant honeybee allergy [6]. The normal baseline tryptase obtained approximately five weeks later does not exclude anaphylaxis because no acute tryptase sample was available [9,10]. Given the severity of the systemic sting reaction, venom immunotherapy was recommended in accordance with current guidance [7,8], but the patient declined treatment after counseling and was advised to return if he wished to reconsider.

The case also highlights the need to manage anaphylaxis and acute coronary syndrome in parallel. Treatment of life threatening anaphylaxis should not be delayed; once myocardial ischemia became evident, aspirin, ticagrelor, intravenous unfractionated heparin, hemodynamic support, and urgent invasive management were added. For future systemic sting reactions, the patient was instructed to use a 0.3 mg epinephrine auto-injector immediately, repeat the dose after 5 to 10 min if needed, and activate emergency medical services. Antihistamines and inhaled salbutamol were prescribed only as adjunctive therapies and should not delay epinephrine [9,10].

The principal limitations are the absence of an acute tryptase sample, the inability to establish the exact temporal origin of the dissection before guide catheter engagement and contrast injection, and the lack of later echocardiography or cardiac magnetic resonance imaging to document infarct extent and functional recovery. Venom-specific IgE establishes sensitization but cannot prove that hypersensitivity caused the coronary dissection, and a single case cannot establish causality or frequency. Overall, the chronology and combined clinical, angiographic, IVUS, echocardiographic, and allergological findings are compatible with suspected type II Kounis syndrome while leaving the mechanism of the dissection unresolved. In addition, only representative stored post-PCI IVUS still frames were available for quantitative reporting; therefore, a pullback-derived minimum stent area, percentage stent expansion, and complete proximal and distal stent edge analysis could not be reconstructed reliably from the static images.

## 4. Conclusions

In this patient, wasp sting anaphylaxis was followed by ST-segment elevation myocardial infarction, with pre-intervention intravascular ultrasound demonstrating a dissection extending from the distal left main coronary artery into the proximal left anterior descending artery. Although a direct causal relationship between the allergic reaction and coronary dissection cannot be established, the chronology and overall clinical findings support suspected type II Kounis syndrome. The case also emphasizes the complementary role of intracoronary imaging in defining lesion morphology and guiding treatment in complex acute coronary presentations associated with hypersensitivity.

## Figures and Tables

**Table 1:** Structured clinical timeline of the index event on 23 June 2026.

Time	Clinical Event, Vital Signs, and Treatment
19:25	Two wasp stings occurred in the left shoulder region. Shortly thereafter, generalized urticaria, facial swelling, dyspnea, loss of consciousness, and hemodynamic instability developed. The exact minute of onset of each individual symptom was not documented.
Before 20:06	At the referring emergency department, intramuscular adrenaline 0.5 mg was administered. Because the clinical response to the first dose was insufficient, a second intramuscular dose of 0.5 mg was given. The exact administration times of the two doses were not documented. Dexamethasone and intravenous fluid resuscitation were also administered.
20:06	Clinical improvement after initial anaphylaxis treatment. Blood pressure 120/80 mmHg, heart rate 65 bpm, respiratory rate 16/min, SpO2 99%.
20:20	Severe pressing chest pain developed, rated 8/10. Blood pressure 105/75 mmHg, heart rate 66 bpm, respiratory rate 20/min, SpO2 99%.
20:28	ECG showed sinus rhythm at 66 bpm with ST-segment elevation in leads I, aVL, and V1 to V6 and reciprocal ST-segment depression in leads II, III, and aVF.
20:35	Chest pain resolved. Blood pressure 110/75 mmHg, heart rate 80 bpm, respiratory rate 20/min, SpO2 99%.
20:39	Repeat ECG showed sinus rhythm at 82 bpm with nonspecific ST and T changes; the previously documented ST-segment elevation had resolved.
20:50	Chest pain recurred, rated 6/10. Blood pressure 120/75 mmHg, heart rate 78 bpm, respiratory rate 18/min, SpO2 99%.
21:00	Chest pain decreased to 1/10. Blood pressure 120/75 mmHg, heart rate 80 bpm, respiratory rate 17/min, SpO2 99%.
21:06	Blood pressure decreased to 90/60 mmHg. Heart rate 82 bpm, respiratory rate 17/min, SpO2 99%.
21:07	Blood pressure 85/60 mmHg, heart rate 70 bpm, respiratory rate 16/min, SpO2 95%. Acute coronary syndrome treatment included aspirin 250 mg orally, ticagrelor 180 mg orally, and unfractionated heparin 5000 IU intravenously. Morphine was administered fractionally.
21:17	Blood pressure remained 85/60 mmHg. An additional 2.5 mg of intravenous morphine was administered, and norepinephrine infusion was initiated.
21:27	Blood pressure decreased to 80/50 mmHg. Norepinephrine infusion was increased; chest pain was controlled.
21:36	Blood pressure improved to 110/70 mmHg, heart rate 72 bpm, respiratory rate 16/min, SpO2 96%.
22:10	Emergency invasive coronary procedure commenced.
23:05	Coronary angiography, IVUS assessment, and PCI of the distal left main to proximal LAD had been completed, including implantation of a Xience Skypoint 5.0 × 15 mm drug-eluting stent.

## Data Availability

The data presented in this case report are available from the corresponding author upon reasonable request. The data are not publicly available due to patient confidentiality and applicable ethical restrictions.

## Supplementary Materials

The following supporting information can be downloaded at https://www.mdpi.com/article/10.3390/jcdd13100509/s1, Video S1: Coronary angiography comprising two initial left coronary projections obtained before guidewire passage, right coronary angiography, and final left coronary angiography after LM to LAD PCI with preserved LCx flow. Video S2: IVUS recording from the index procedure demonstrating the distal left main to proximal LAD culprit morphology, including the intimal flap and dissection plane before stent implantation.
